# Training Community Health Workers for Diabetes Management in Low- and Middle-Income Countries: Systematic Review

**DOI:** 10.2196/84508

**Published:** 2026-06-10

**Authors:** Anirudh Gaurang Gudlavalleti, Sureshkumar Kamalakannan, Sophie Vanbelle (S), Venkata Satyanarayana Murthy Gudlavalleti, Nicolaas C Schaper, Giridhara R Babu, Onno CP van Schayck

**Affiliations:** 1 Department of Family Medicine, Care and Public Health Research Institute, Maastricht University, Maastricht, The Netherlands; 2 Department of Public Health, Pragyaan Sustainable Health Outcomes Foundation, Level 2, Kapil Kavuri Hub, Financial District, Nanakramguda, Hyderabad, 500032, India, 91 8008799816; 3 Department of Social Work, Education and Community Wellbeing, Northumbria University, Newcastle Upon Tyne, United Kingdom; 4 Methodology and Statistics, Faculty of Psychology and Neuroscience, Maastricht University, Maastricht, The Netherlands; 5 Department of Internal Medicine, Maastricht University, Care and Public Health Research Institute (CAPHRI), Cardiovascular Research Institute Maastricht (CARIM), Maastricht, The Netherlands; 6 Department of Population Medicine, College of Medicine, QU Health, Qatar University, Doha, Qatar

**Keywords:** community health workers, community health workers training, diabetes management, task shifting, systematic review, low- and middle-income countries, LMICs, glycated hemoglobin, HbA_1c_

## Abstract

**Background:**

Type 2 diabetes mellitus, a public health challenge, disproportionately impacts low- and middle-income countries (LMICs), accounting for 73% of global cases. Due to resource constraints, these nations have adopted task-shifting strategies using community health workers (CHWs). However, evidence on the effectiveness of training CHWs in diabetes management is limited and, at most, indirect due to the limited studies, variable training methods, and complex interventions that make it difficult to isolate training effects.

**Objective:**

A systematic review was conducted to answer the question: Does training CHWs in type 2 diabetes improve the efficacy of diabetes screening and management at the community level in LMICs?

**Methods:**

A total of 2 reviewers, supervised by 2 supervisors, conducted the review following the PRISMA (Preferred Reporting Items for Systematic Reviews and Meta-Analyses) 2020 guidelines. They searched databases, including PubMed, MEDLINE Ovid, Scopus, EBMR, and CINAHL, for studies published between January 2000 and April 2024, including randomized and nonrandomized controlled trials and observational studies assessing CHW training in diabetes management in LMICs. The primary outcome was the mean change in glycated hemoglobin (HbA_1c_) percentage levels. Data were narratively synthesized for training characteristics and study outcomes, and quality was assessed using the Risk of Bias 2 and ROBINS-I tools.

**Results:**

A total of 3387 studies were screened; 69 were eligible for full-text review, and 4 studies (3 randomized controlled trials [RCTs] and 1 observational stepped-wedge study, ~1000 patients) were included for narrative analysis. One of the 3 RCTs reported a statistically significant mean HbA_1c_ reduction of −0.24% (*P*=.001), but HbA_1c_ was not the primary outcome, and most patients were normoglycemic, prediabetic, or had diabetes. Other studies reported nonsignificant HbA_1c_ reductions. The risk of bias among RCTs was moderate (some concerns, 1 trial at high risk), and the observational study had a serious risk of bias. No meta-analysis was performed due to the limited number of RCTs.

**Conclusions:**

Training CHWs in type 2 diabetes management has shown limited and, at most, indirect effects in improving glycemic control in LMIC settings. These findings are constrained by the small number of eligible studies, heterogeneity in training methodologies, and the multicomponent nature of the included interventions, with 1 trial demonstrating a statistically significant yet small reduction in HbA_1c_ (−0.24%). Our review included only 4 eligible studies with a small representation of CHWs and multicomponent interventions. Considering the limited number of eligible studies, the heterogeneity in training methodologies and study designs, and the multicomponent nature of the included interventions, the existing evidence remains inadequate to definitively conclude whether CHW training significantly improves diabetes management across LMICs. Therefore, strengthening and standardizing CHW training might be an effective strategy to enhance diabetes care in underserved settings. Future larger trials and implementation research can help maximize the impact of CHWs against the growing diabetes burden.

## Introduction

### Rationale

Diabetes mellitus is a growing pandemic in the global community, with 589 million people affected by it [1], while also representing one of the leading factors of mortality and morbidity globally [2]. Nearly 430 million people with diabetes, which is 73% of such patients, reside in low- and middle-income countries (LMICs) [1]. The steadily rising prevalence [3] imposes a substantial burden on health care systems due to the complexity of accessing health care in these countries [4-6]. Furthermore, individuals residing in rural areas encounter numerous barriers to accessing health care, which can be broadly categorized into 4 key areas: geographical accessibility, health care availability, financial accessibility, and the acceptability of care. Geographical accessibility refers to the ease or difficulty of reaching health care facilities, which is more pronounced in rural areas. This is due to the large distances rural patients must traverse and the limited transportation infrastructure. Health care access is limited by shortages of facilities and trained staff, especially in rural areas. Financial barriers, particularly out-of-pocket costs, prevent many people, especially those from lower socioeconomic groups, from accessing health care. Additionally, sociocultural factors like mistrust or stigma can deter patients from seeking care. These barriers disproportionately affect rural populations and those from lower socioeconomic strata [2,7-12].

Many LMICs employ local community health workers (CHWs) to improve access to health care. CHWs are usually individuals from the communities they serve, selected by those communities, and accountable to them for their work. While CHWs should receive support from the health system, they are not required to be formally integrated into its organizational structure. Additionally, their training is typically shorter than that of professional health care workers [13]. Referred to as task shifting, using CHWs for disease management is effective for infectious diseases (eg, HIV and hepatitis), especially in pregnant women, newborns, and mothers [14,15], and for noncommunicable diseases, like diabetes and cardiovascular diseases [16-25]. While most LMICs consider skilling and implementing task shifting using CHWs to improve care quality [20], they are limited by a lack of clarity on the exact nature, content, and skills of the CHWs. Evidence suggests that the quality of health care at the primary care level is associated with the limited knowledge and requisite skills among CHWs. Hence, most task-shifting initiatives also focus on imparting requisite training to CHWs [9,26-32]. Despite many CHW training programs, there is limited systematic evidence of their impact on diabetes outcomes in LMICs. While some reviews have examined CHW roles in diabetes globally, none have specifically focused on the effect of CHW training in LMIC contexts. Despite this, the evidence regarding the impact of CHW training in LMICs is still limited and mostly indirect, and no systematic review has specifically addressed this issue. Hence, this paper aims to systematically review the available evidence to answer the question: Does training CHWs in diabetes improve the efficacy of diabetes screening and management at the community level in LMICs?

### Objective

We hypothesize that CHW training will improve glycemic control among patients managed by the trained CHWs. By identifying and synthesizing existing studies, this review also aims to assess whether training can be a necessary but insufficient component of a multicomponent intervention for effective CHW-led diabetes management, highlighting knowledge gaps and informing future improvements in community-based diabetes care. This review also aims to identify gaps in the reporting of CHW training.

## Methods

### Study Design and Registration

We followed the PRISMA (Preferred Reporting Items for Systematic Reviews and Meta-Analyses) 2020 guidelines (Checklist 1) [33,34]. The review was registered with PROSPERO (CRD42022341717) and was published previously [35].

### Eligibility Criteria

Randomized controlled trials (RCTs), non-RCTs, and observational studies that assessed the training of CHWs in the management of type 2 diabetes mellitus were included. The CHWs were included based on the definition provided by the World Health Organization (WHO) [36]. The PICOS (population, intervention, comparator, outcome, and study setting) framework was used to finalize the specific inclusion criteria, which were as follows:

- Population: the population included was CHWs managing persons with type 2 diabetes from LMICs. The WHO definition of CHWs was used, which defines them as “members of the communities where they work, should be selected by the communities, should be answerable to the communities for their activities, should be supported by the health system but not necessarily be a part of its organization, and have shorter training than professional workers” [13]. Only studies in which the target population was 18 years or older were included. The baseline qualifications of the CHWs were not reported in the included studies.
- Intervention: the review included any study in which CHWs received training in diabetes management, focusing on their knowledge, skills, and practices in diabetes management in LMICs, including blood glucose monitoring, lifestyle modification, and prevention of complications. All the included studies were implemented in a multicomponent nature, wherein they combined training with other components of the intervention, and only one of them (Catley et al [37]) isolated the intervention effect. Thus, the intervention’s (training’s) effects could not be isolated from the delivery intensities of the interventions reported in the included studies.
- Comparator: standard diabetes care, no intervention, or other training programs unrelated to diabetes served as comparators.
- Outcomes: the primary outcome of interest was the mean change in the glycated hemoglobin (HbA_1c_) levels of individuals with diabetes managed by the CHWs. HbA_1c_ values were included even when reported as secondary outcomes in a study. The secondary outcomes of interest were other clinical measures, such as random and fasting blood glucose, lipid profile, total cholesterol, and blood pressure (systolic and diastolic). A study was included only when HbA_1c_ levels were reported. HbA_1c_ was chosen as the primary clinical outcome of interest as it is the gold standard recommended by the WHO and the International Diabetes Federation, as it reports the glycemic control of the patient over a 3-month period. However, limiting the analysis to only this outcome would have excluded studies using alternative clinical outcomes, thereby limiting our conclusions regarding the effect of CHW training on diabetes outcomes. Additionally, our research question encompassed diabetes screening along with management, but no eligible studies reporting screening outcomes were identified.
- Study setting: studies conducted in LMICs, as defined by the World Bank, were included (countries with a gross national income per capita between US $1136 and US $4465) [38].
- Exclusion criteria: we excluded studies that did not have CHWs as defined by the WHO; studies that did not train CHWs; studies that did not report HbA_1c_ as an outcome; studies that were not conducted in LMICs; studies that were protocols, reviews, qualitative studies, editorials, and conference abstracts; and studies that were not published in English.

It is important to note that CHW capacity building and service delivery exposure are distinct components of the intervention. Capacity building refers to the structured training interventions provided to the CHWs, with diversity in the curriculum, pedagogy, mode of training, and frequency of training. The service delivery component refers to the activities concentrating on the contact between the CHWs and the patients, such as the type of contact, clinical monitoring, and paraclinical support. Thus, while the capacity building component focuses on CHW competency, the service delivery component focuses on postintervention implementation. Both of these components together represent the effectiveness of the intervention.

### Search Strategy

A comprehensive search was conducted across the following databases: PubMed, MEDLINE Ovid, Scopus, EBMR, and CINAHL, spanning the period from January 2000 to April 2024. This period was chosen because the World Medical Association and the WHO published their respective task-shifting guidelines using CHWs around this time [39,40]. The search was limited to studies published in the English language. It used MeSH terms and free text related to CHWs, type 2 diabetes, training, and outcome measures such as HbA_1c_, random blood glucose, oral glucose tolerance test, and microvascular complications. Additional searches were conducted in gray literature databases, including the WHO International Clinical Trials Registry Platform and ClinicalTrials.gov. The detailed search strategy is provided in Annexure 2 in Multimedia Appendix 1.

### Study Selection and Data Extraction

A total of 2 reviewers (AGG and SK) independently screened the titles and abstracts of studies that might meet the inclusion criteria. Data were retrieved for full-text review, and those that fulfilled the inclusion criteria were extracted. A third reviewer (GRB) resolved any disagreements.

Data extraction was conducted using a customized version of the standardized form developed in Covidence (Veritas Health Innovation) software. An initial preliminary round of data extraction was performed with 4 studies each by AGG and SK. Once their interreviewer consistency was confirmed, further extraction was carried out. The data collected included study characteristics (author, year, country, and sample size), details of the intervention (CHW training content, duration, and frequency), and outcome measures (mean change in HbA_1c_, expressed as a percentage, and secondary outcomes). Additionally, all training-related data points were extracted, including the contents of the training module, mode of delivery, duration, and frequency of training and refresher sessions (if applicable). This was done to assess whether any of these factors were related to our outcome of interest and, if so, to what extent. All data were organized into tables for narrative comparison among the studies.

### Quality Assessment

The quality of the included studies was assessed using the second version of the Cochrane Risk of Bias 2 (RoB 2) tool [41] for RCTs and the ROBINS-I tool [42] for nonRCTs. The RoB 2 tool evaluated RCTs across 5 domains: bias from randomization, deviations, missing data, outcome measurement, and reporting. The ROBINS-1 tool assessed nonRCTs across 7 domains: bias from confounding, participant selection, intervention classification, deviations, missing data, outcome measurement, and reporting. Each domain was scored as follows: low risk, some concerns, moderate risk, serious risk, and critical risk. Additionally, the masking of the assessors was not reported in the included studies, so it is not clear whether these studies were (partly) blinded or not.

### Data Synthesis and Analysis

The data from all of the included studies were extracted using forms we developed with the Covidence software. All data were exported into Microsoft Excel (Office 2021). The studies were subsequently analyzed narratively, considering the aim and objectives, the inclusion and exclusion criteria, the training characteristics, the outcome values for HbA_1c_, and the strengths and limitations of each study. We summarized the findings descriptively in text and a comparative table, recording which training features seemed most aligned with improved glycemic control. This approach enabled us to explore thematic links between specific training modalities and HbA_1c_ outcomes without formally combining effect sizes into a single quantitative estimate. A meta-analysis was not performed in accordance with the Cochrane Handbook’s recommendation against conducting a meta-analysis with a small number of studies, as the pooled evidence may report unreliable precision and heterogeneity among the studies [43]. The heterogeneity among the studies was conceptual and intervention-based rather than statistical.

## Results

**Figure 1.**
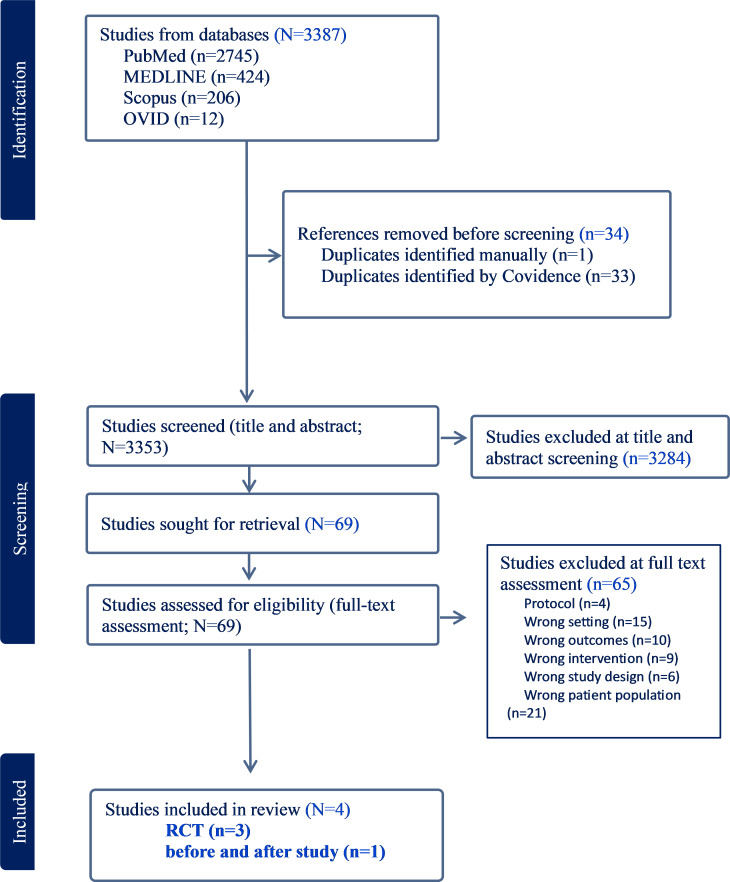
PRISMA (Preferred Reporting Items for Systematic Reviews and Meta-Analyses) 2020 flowchart diagram: Interventions involving community health workers training for diabetes in low- and middle-income countries.

We identified 3387 studies for abstract screening, of which 69 were found eligible for full-text review (Figure 1). Upon further screening, 4 studies conducted in LMICs were included, all of which involved the assessment of the impact of training CHWs on the mean reduction of HbA_1c_ levels. However, none of these studies reported any diabetes screening outcomes. Table 1 presents the study characteristics, training characteristics, sample sizes, and primary and secondary outcomes.

**Table 1.** Study characteristics of interventions involving community health worker training for diabetes in low-income and middle-income countries.

Study	Study design	Number of CHWs^a^ trained	CHW training	Participant characteristics	HbA_1c_%^b^			HbA_1c_ outcome	Follow-up
					Arm comparison at follow-up	Change within arms	Arm comparison at follow-up		
Jain et al [44]	Open-label RCT^c^ 6 months follow-up Maharashtra, India	Intervention: 2 new CHWs Control: NM^d^	7-day workshop on communication, adherence, complication recognition, and monitoring of diabetes	Patients with type 2 diabetes	Intervention (n=151): 7.63 (SD 2.16) Control (n=139): 7.64 (SD 1.79) (at 6 mo)	Intervention: *P*<.01 Control: *P*<.01 Statistical test: paired *t* test	*P*=.95 Statistical test: ANCOVA adjusting for baseline	Primary	6
Catley et al [37]^e^	Cluster RCT 7‐9 months follow-up Cape Town, South Africa	NM	3-day workshop and 8 weekly half-day experiential sessions on motivational interviewing and behavior-change techniques.	Patients with BMI≥25 kg/m^2^ support group members receiving health services from local nongovernmental organizations Intervention (n=240): normoglycemia (n=65, 27.1%), prediabetes (n=106, 44.2%), diabetes (n=69, 28.8%) Control (n=254): normoglycemia (n=62, 24.4%), prediabetes (n=135, 53.1%), diabetes (n=57, 22.4%).	Intervention (n=215): 6.32 (SD 1.34); normoglycemia Control (n=223): mean 6.38 (SD 1.54), normoglycemia (n=64, 28.7%), prediabetes (n=106, 47.5%), and diabetes (n=53, 23.8%).	Intervention: NM Control: NM	Adjusted mean difference: –0.24 (SD 0.08) *P*=.001 Statistical test: multilevel ANCOVA fixed effects: arm and baseline value	Secondary	7‐9
de Souza et al [45]	RCT; 3 months follow-up Conducted in São Pedro, Brazil	Intervention: 4 new CHWs Control: 4 new CHWs	Intervention: 4 weekly 60-min diabetes sessions on diabetes definition, lifestyle therapy, pharmacological options, and chronic complications. Control: 4 classes on tuberculosis, asthma, and contraception. All CHWs made monthly home visits	Patients with type 2 diabetes are included in CHW care	Intervention (n=62): mean 7.9 (SD 1.9) Control (n=56): mean 8.4 (SD 2.5)	Intervention: *P*<.001 Control: *P*<.001	*P*=.13 Statistical test: Student *t* test (HbA_1c_), repeated-measures ANOVA, and log-transform for skewed TG^f^	Primary	3
Worster et al [46]	Observational stepped-wedge study with quarterly follow-up for 12 months Conducted in Chiapas, Mexico	NM	1 month, 16-session course with monthly refreshers on pathophysiology, motivational interviewing, and home visit logistics. The CHWs received refresher training throughout the 12-month period.	Patients (>18 y) diagnosed with and being treated for diabetes and/or hypertension	N/A^g^	–0.35% (95% CI –0.90 to 0.20); *P*=.21	Linear mixed-effects (random intercepts for person and community)	Primary (for patients with diabetes)	12

a CHW: community health workers.

b HbA_1c_: glycated hemoglobin.

c RCT: randomized controlled trial.

d NM: not mentioned.

e Catley et al is the only study to report a statistically significant HbA_1c_ difference between the arms [37].

f TG: triglycerides.

g N/A: not applicable.

Overall, 4 studies involving a total of at least 10 CHWs and around 1000 patients were eligible for inclusion, of which 3 were RCTs [37,44,45] and one was an observational stepped-wedge study [46]. We observed that the training varied in content, delivery, duration, frequency, and settings (Table 1). However, some of the content overlapped with common topics, such as diabetes management, lifestyle modification, and patient education strategies.

Jain et al [44] conducted an open-label RCT in rural Maharashtra, India, with 299 patients with type 2 diabetes: 153 in the intervention arm (trained CHWs) and 146 in the control arm. At the 6-month follow-up, 290 participants responded: 151 from the intervention group and 139 from the control group. A total of 2 CHWs were trained in a 7-day workshop, carried out for home visits and 12 phone reminders over 6 months. Results reported no significant between-arm differences in HbA_1c_ at 6 months (*P*=.51). Both arms showed HbA_1c_ from baseline (intervention: mean 7.63%, SD 2.16% vs control: mean 7.64%, SD 1.79%; *P*=.95; Table 1). Similarly, no differences were found for glucose, blood pressure, low-density lipoproteins, high-density lipoproteins, or triglycerides. The RoB 2 assessment indicated some concerns, and the number of CHWs included in the intervention arm was very small (n=2); the number in the control arm was not reported.

The study by Catley et al [37] was a cluster-RCT conducted in Cape Town suburbs, assessing weight loss as the primary outcome and HbA_1c_ changes as a secondary outcome. Around 28 clusters were randomized between the intervention group (Lifestyle Africa) and the control group. The intervention, Lifestyle Africa, was adapted from the Diabetes Prevention Program, a lifestyle modification program targeting weight loss through motivational interviewing and behavior change methods. Out of 494 enrolled overweight or obese adults, 438 completed the 7 to 9 month follow-up: 215 in the intervention group and 223 in the control group. Among the enrolled participants, 28.8% (69/240) in the intervention arm and 22.4% (57/254) in the control arm had diabetes. The intervention group included 27.1% (65/240) adults with normal glucose metabolism status, 44.2% (106/240) with prediabetes, and 28.8% (69/240) with diabetes; these categories were based on HBA_1c_. The control group had 24.4% (62/254) adults with normal glucose metabolism, 53.1% (135/254) with prediabetes, and 22.4% (57/254) with diabetes. The CHWs attended a 3-day workshop and 8 sessions featuring 17 videos on motivational interviewing and behavior change. CHWs role-played as mock participants, practicing activities such as weighing participants, using video equipment, and facilitating discussions. Participants in the intervention arm (Lifestyle Africa) experienced a significant reduction in HbA_1c_ compared to the control group (adjusted mean difference –0.24%; *P*=.001). No significant changes were observed in the primary outcomes, including blood pressure, low-density lipoproteins, high-density lipoproteins, triglycerides, and cholesterol levels. Participants in the intervention arm had a higher probability of improving and a lower probability of worsening their glucose metabolism status by at least 1 category (odds ratio [OR]=1.52, 95% CI 1.04-2.22; *P*=.03). The overall RoB 2 assessment indicated some concerns. Therefore, the HbA_1c_ reduction reported reflects a shift in the overall glycemic risk of the overweight adult population in the study and not a trial designed to test the efficacy of diabetes management among patients with diabetes.

The study by de Souza et al [45] was a randomized controlled trial conducted in Porto Alegre, Brazil. The study aimed to measure the percentage change in mean HbA_1c_ between intervention and control groups. A total of 118 patients were randomly allocated to 8 CHWs, with 4 trained in diabetes through 4 weekly 60-minute sessions (covering diabetes definition, lifestyle, medications, and complications) in the intervention arm, which included 62 patients, and 4 in the control arm, which included 56 patients, trained in unrelated health topics such as tuberculosis, asthma, and contraception. All CHWs made monthly home visits. At the 3-month follow-up, there was no statistically significant difference in the percentage of mean HbA_1c_ between the arms (intervention: mean 7.9%, SD 1.9%; control: mean 8.4%, SD 2.5%; *P*=.13). Total cholesterol and triglyceride levels declined more in the intervention group, whereas blood pressure and high-density lipoprotein levels changed similarly in both groups. The overall RoB 2 score indicated high risk, and the number of CHWs in each arm was relatively small (n=4 in each arm).

The study by Worster et al [46] was an observational study conducted in the communities of Chiapas, Mexico. It aimed to study changes in diabetes and hypertension prevalence among patients through CHWs in primary care settings. In total, 149 patients were included, with 73 treated for diabetes. The study followed an observational stepped wedge design over 12 months, with data collection at baseline and every 3 months for 12 months after the implementation of the intervention with newly trained CHWs. In the subgroup of participants with diabetes and a baseline HbA_1c_≥9%, this value significantly decreased by ~0.96% (*P*=.01), but for all patients with diabetes, the mean decrease (~0.35%) was not statistically significant (*P*=.21). The risk of bias was assessed as serious using the ROBINS-I tool. (The detailed scores with explanations of the various domains for both RoB-2 and ROBINS-I tools are shown in Annexure 3 in Multimedia Appendix 2).

## Discussion

### Principal Findings

The results of our systematic review show that improvement in HbA_1c_ (at the population level) was moderate and statistically significant in only 1 study by Catley et al [37]. Since HbA_1c_ was not the primary outcome of interest among its sample of individuals with normoglycemia, prediabetes, and diabetes, our review concludes that the evidence for training CHWs in diabetes management in LMICs is limited and, at most, indirect. The limited number of eligible studies, the heterogeneity in training methodologies (ranging from 4-hour sessions to multiday workshops), study designs, and the multicomponent nature of the included interventions limit the interpretation of our results. Therefore, the existing evidence remains inadequate to definitively conclude whether CHW training significantly improves diabetes management across LMICs.

The Catley et al’s [37] study evaluated “Lifestyle Africa,” an adapted version of the Diabetes Prevention Program, a structured lifestyle modification program that primarily targeted weight loss through motivational interviewing and behavior change techniques. The study involved CHW-patient contact after CHW training, with nearly 29 sessions (17 core and 12 postcore sessions) and a maximum follow-up period of 12 months (varying from 3 to 12 months), thereby providing sufficient time to detect a significant difference in HbA_1c_ levels. However, the study included patients who were normoglycemic, prediabetic, and diabetic, and the change in HbA_1c_ values (a secondary outcome) was recorded for this total population. Thus, the results may not be attributed directly to people with diabetes or generalized to the subgroup of people with only diabetes. The other 2 RCTs (Jain et al [44] and de Souza et al [45]) showed a reduction in HbA_1c_ levels within both arms, but no difference was observed between the study arms. Both studies had shorter CHW-patient interactions (16 contacts and 3 contacts, respectively) and shorter follow-up periods (6 mo and 3 mo, respectively). Meanwhile, the observational study by Worster et al [46] documented a statistically significant change in the percentage of mean HbA_1c_ levels only in patients with HbA_1c_>9%. However, this could have been due to the regression-to-the-mean effect [47], which may have arisen due to the selection of patients with extreme HbA_1c_ values. In addition, the use of unadjusted statistical tests, like the Student *t* test, could have potentially overestimated the actual effect of the intervention. All included studies showed varying bias levels, affecting interpretation. RCTs by Jain et al [44] and Catley et al [37] had some concerns, the RCT by de Souza et al [45] had high bias, and the observational study by Worster et al [46] was rated serious. These ratings highlight the possibility of systematic errors affecting the reliability of and confidence in conclusions derived from the intervention outcomes.

Regarding treatment in the control arms, the participants in the studies by Jain et al [44] and de Souza et al [45] received enhanced usual care, which could have led to changes in behavior and reduced HbA_1c_ levels in the control arms of these studies. Specifically, in the study conducted by Jain et al [44], participants in the control group attended regular physician clinics and interacted frequently with their doctors. In the study by de Souza et al [45], although the CHWs in the control arm were not explicitly trained in diabetes management, they still conducted monthly home visits addressing other health topics (eg, maternal health, immunization, tuberculosis, and asthma), thereby ensuring consistent personal contact. These activities introduce 2 well-documented mechanisms for glycemic improvement. First, repeated contact, measurement, and observation can enhance patients’ self-monitoring and adherence to lifestyle advice (the Hawthorne effect), even when the advice is unrelated to diabetes. Second, regular supportive contact (nondiabetes-focused contact) from a trusted CHW or physician can provide nonspecific therapeutic exposure, improving self-efficacy, medication adherence, and problem-solving behaviors, which are known to lower HbA_1c_ [47,48]. Therefore, reductions seen in the control arms are expected to be probable and reasonable, potentially attenuating the intervention effect size.

The outcome variation across studies may result from several factors, primarily due to varying intensity, duration, and content of the training programs for the CHWs. The trial with significant HbA_1c_ benefit (Catley et al [37]) involved a 3-day workshop and 8-day follow-up sessions with ongoing support via videos and SMS text messaging for CHWs spanning 7‐9 months. The study’s training emphasized practical skills, such as motivational interviewing and behavior-change techniques, to enhance patient engagement and coaching. In contrast, the trial by de Souza et al [45] featured a short, single 4-hour training session that emphasized basic diabetes knowledge but lacked ongoing mentorship, leading to no meaningful improvements in glycemic control. These trends may suggest a dose-response relationship, where more intensive, skill-focused training of CHWs improves patient outcomes. These findings imply that CHW training interventions can potentially improve diabetes outcomes, particularly in subpopulations with uncontrolled glycemia; however, consistent and significant effects were not observed across all settings. Published evidence from higher-income countries and from peer-led interventions using CHWs has demonstrated the success of these interventions, advocating for their trial and implementation in LMICs, where a shortage of rural doctors is prevalent. The systematic review by Werfalli et al [49] of peer or CHW-led self-management support in LMICs (where patients managed their diabetes with CHW guidance) reported a statistically significant reduction in HbA_1c_ across studies. Meanwhile, the review by Palmas et al [50] reported modest HbA_1c_ reduction against standard care. The review by Shah et al [51] reported comparable results. Similarly, the DIABLEST trial by Pérez-Escamilla et al [52] and the study by Aponte et al [53] conducted in the United States also reported a statistically significant change in the HbA_1c_ percentage for CHW-led groups compared to the study’s control arm. These studies were excluded from our review due to their intervention or location not satisfying our inclusion criteria. Recently, the systematic review by Evans et al [54] reported positive effects across a broader set of studies [54]. Our review differed in scope since we focused on CHW training as the intervention for LMICs and HbA_1c_ as the inclusion criterion. The findings from Evans et al [54] and our review highlighted that, while CHW involvement in diabetes care may be beneficial, the specific and independent contribution of CHW training in LMICs to diabetes care requires further investigation.

The United Kingdom Prospective Diabetes Study [55] showed that a modest reduction in HbA_1c_ levels can be associated with meaningful clinical benefits, such as lowering the risk of future diabetic complications in the eyes, kidneys, and nerves. Even a 1% decrease in HbA_1c_ levels can result in a 37% reduction in combined microvascular complications (*P*<.001), a 21% reduction in risk for any endpoint related to diabetes (*P*<.001), and a 21% reduction in deaths related to diabetes (*P*<.001) [55,56]. Thus, even the approximately 0.3% to 0.5% HbA_1c_ reductions observed in one of our included studies could translate into clinically important benefits. We did not analyze the secondary outcomes, as there was variation in the measurement and reporting of the outcomes across the three RCTs.

### Strengths and Limitations

The study has several strengths. This review is one of the first to outline the advantages of training CHWs for improved glycemic control over generic methods used in LMICs. The review also establishes robust methodological rigor, supported by a registered protocol, a PRISMA-guided screening process, and a formal Risk of Bias (RoB) 2 and ROBINS-I appraisal. By integrating a 24-year multidatabase search with pooled and narrative analyses on training intensity and content, this review provides insights relevant to policy and scalable within task-shifting programs.

This study faced limitations, including variability among the included studies in design, intervention, and outcomes. The follow-up periods of all studies varied considerably, ranging from 3 to 12 months. Our focus on LMICs could have narrowed the evidence base while not reflecting the success of CHW training in high-income countries. Thus, the results need to be interpreted cautiously and might not be generalizable. With only 4 trials, testing for publication bias was not performed [57]. Three studies did not isolate the intervention effects of CHW training from other intervention components, so HbA_1c_ changes reflect combined effects, not just training. Only Catley et al [37] isolated the effect of lifestyle coaching statistically. Other studies risked performance and Hawthorne effects due to additional contact and training, possibly blurring causality. Small sample sizes in Jain et al [44] and de Souza et al [45] may also limit external validity. The scale-up of programs with diverse CHWs may overestimate effects. The implementation of skills gained by CHWs was not reported, which is crucial. Overall, the evidence quality has concerns due to biases and deviations. The certainty of the evidence was low to moderate, with concerns surrounding performance bias that might have introduced Hawthorne effects. The high risk of bias in de Souza et al [45], along with concerns about the randomization process, raises questions about the equal distribution between the arms at baseline. The serious risk of bias in Worster et al [46] due to confounding and deviations from the interventions also raises questions about causal inference. All interventions were multicomponent, so individual effects cannot be separated. Despite extensive searches, unpublished studies might have been missed, risking publication bias.

### Implications

While results across RCTs might suggest potentially beneficial effects—despite limitations, with only 1 study showing a significant effect—of introducing CHWs in diabetes care in LMICs, the small number of trials and their limitations preclude the drawing of firm conclusions. Therefore, we suggest that training characteristics, subsequent community interventions, and data collection and analyses must be studied further to develop an optimal training program for CHWs to manage diabetes and monitor the performance of the CHWs at the community level. In addition, such trials should be sufficiently powered, with adequate numbers of both CHWs and patients included. Additional trials, designed to isolate the impact of CHW training from other intervention components, such as service delivery intensity and CHW-patient contact frequency, are necessary to thoroughly establish a link between CHW training and diabetes care in LMICs. Finally, we realize that many studies may not have met our stringent eligibility criteria, and thus, the results cannot be generalized. We therefore propose that future scoping reviews be carried out to address this shortcoming.

This review highlights significant implications for health care practice and policy in LMICs. CHWs are essential for improving health care delivery to underserved groups. Offering them contextually relevant, standardized, and comprehensive training, along with ongoing support and periodic refresher sessions, can help sustain and boost the effectiveness of these interventions over time. Policymakers and health leaders should consider incorporating structured diabetes management training into CHW curricula and conducting regular reviews and refresher courses to maintain their skills. Future research should focus on identifying which CHW training elements have the greatest impact. Larger RCTs with more CHWs and standardized outcomes beyond just HbA_1c_ are necessary to better assess specific component efficacy. Moreover, qualitative studies exploring the experiences and challenges of CHWs in implementing diabetes care can offer valuable insights into improving training programs and support systems, complementing the findings from RCTs.

### Conclusions

In conclusion, our review suggests that structured CHW training may be an avenue to improve diabetes outcomes in low-resource settings, but current evidence is insufficient, and more robust scientific evidence is needed before firm conclusions can be drawn. Moreover, its implementation should be accompanied by rigorous evaluation to assess and optimize its impact.

## Supplementary material

10.2196/84508 Multimedia Appendix 1 Annexure-2: search strategies.

10.2196/84508 Multimedia Appendix 2 Annexure-3: risk of bias.

10.2196/84508 Checklist 1 PRISMA checklist

## Abbreviations

CHW: community health worker
HbA_1c_: glycated hemoglobin
LMIC: low- and middle-income country
PICOS: population, intervention, comparator, outcome, and study setting
PRISMA: Preferred Reporting Items for Systematic Reviews and Meta-Analyses
RCT: randomized controlled trial
WHO: World Health Organization
