# Supplementary material for: Training Community Health Workers for Diabetes Management in Low- and Middle-Income Countries: Systematic Review
Source: JMIR Diabetes. 2026 Jun 10;11:e84508. doi: 10.2196/84508 (PMC13252886; doi:10.2196/84508)
Supplement: Multimedia Appendix 1 [file diabetes-v11-e84508-s001.pdf]

Annexure-2: Search Strategies

PubMed

((community health worker[MeSH Terms]) OR (community health workers[MeSH Terms])) OR (lay worker))) OR (CHW)) OR (health worker)) OR (health worker, community[MeSH Terms])) OR (health worker, village[MeSH Terms])) OR (health workers, community[MeSH Terms])) OR (ASHA)) OR (ANM)) OR (MPHW)) OR (JHA)) OR (MPW)) OR (MPHA)) AND (academic training[MeSH Terms])) OR (traini[MeSH Terms])) OR (activities, training[MeSH Terms])) OR (training)) OR (capacity building[MeSH Terms])) OR (activities, educational[MeSH Terms])) OR (activity, educational[MeSH Terms])) OR (education)) OR (hospital, teaching[MeSH Terms])) OR (hospitals, teaching[MeSH Terms])) OR (method, teaching[MeSH Terms])) OR (teaching)) OR (interactive tutorial[MeSH Terms])) OR (tutoring)) OR (coaching)) OR (supervision[MeSH Terms])) AND (effect, placebo[MeSH Terms])) OR (effects, placebo[MeSH Terms])) OR (placebo effect[MeSH Terms])) OR (placebos[MeSH Terms])) OR (placebo effects[MeSH Terms])) OR (care standard[MeSH Terms])) OR (care standards[MeSH Terms])) OR (standard training)) AND (type 2 diabetes mellitus[MeSH Terms])) OR (Typ[MeSH Terms])) OR (blood glucose[MeSH Terms])) OR (home blood glucose monitoring[MeSH Terms])) OR (monitoring, home blood glucose[MeSH Terms])) OR (Fasting Blood Glucose)) OR (FBS)) OR (Random Blood Glucose)) OR (Random Blood Sugar)) OR (Fasting Blood Sugar)) OR (RBS)) OR (consultation and referral[MeSH Terms])) OR (hospital referral[MeSH Terms])) OR (hospital referrals[MeSH Terms])) OR (referrals, hospital[MeSH Terms])) OR (referrals[MeSH Terms])) OR (physician self referral[MeSH Terms])) OR (physician self referrals[MeSH Terms])) OR (referrals, hospital[MeSH Terms])) OR (self referrals, physician[MeSH Terms])) OR (screening))) OR (a1b hemoglobin, glycosylated[MeSH Terms])) OR (glycosylated a1b hemoglobin[MeSH Terms])) OR (glycosylated hemoglobin[MeSH Terms])) OR (HbA1c)) OR (ogtt[MeSH Terms])) OR (glucose tolerance test[MeSH Terms])) OR (glucose tolerance tests[MeSH Terms])) OR (glucose tolerance, oral[MeSH Terms])) OR (microvascular complications)) OR (DR Screening)) OR (Diabetic Retinopathy Screening)) OR (Peripheral Neuropathy Screening) AND (clinicaltrial[Filter] OR meta-analysis[Filter] OR randomizedcontrolledtrial[Filter])) AND (countries, developing[MeSH Terms])) OR (countries, developing[MeSH Terms])) OR (countries, less developed[MeSH Terms])) OR (LMIC)) OR (Low and Middle Income Country)) OR (Low and Middle Income Countries) Filters: Clinical Trial, Clinical Trial, Phase III, Controlled Clinical Trial, Meta-Analysis, Pragmatic Clinical Trial, Randomized Controlled Trial, Systematic Review, Adult: 18+ years, from 2000/1/1 - 2024/4/1

Scopus

(TITLE-ABS-KEY ("community health worker" OR "community health workers" OR CHW OR "lay health worker" OR "health worker" OR "village health worker" OR ASHA OR ANM OR MPHW OR JHA OR MPW OR MPHA)

AND TITLE-ABS-KEY (training OR "academic training" OR "capacity building" OR education OR teaching OR supervision OR coaching OR tutorial OR tutoring)

AND TITLE-ABS-KEY ("type 2 diabetes mellitus" OR "blood glucose" OR HbA1c OR "glycosylated hemoglobin" OR "fasting blood glucose" OR "random blood glucose" OR "glucose tolerance test" OR OGTT OR "diabetic complications" OR "microvascular complications")

AND TITLE-ABS-KEY ("clinical trial" OR "randomized controlled trial" OR "meta-analysis" OR "systematic review")

AND TITLE-ABS-KEY ("low and middle income country" OR "low and middle income countries" OR LMIC OR "developing country" OR "developing countries")

AND PUBYEAR > 1999 AND PUBYEAR < 2025

Medline (OVID)

1 community {No Related Terms} (39719)

2 health worker {No Related Terms} (6629)

3 1 and 2 (377)

4 lay health worker {No Related Terms} (3986)

5 community health worker {No Related Terms} (1233)

6 CHW {No Related Terms} (1452)

7 4 or 5 or 6 (5764)

8 4 and 5 and 6 (194)

9 diabetes mellitus {No Related Terms} (10248)

10 type 2 diabetes {No Related Terms} (15087)

11 diabetes {No Related Terms} (13755)

12 T2DM {No Related Terms} (3887)

13 DM {No Related Terms} (3275)

14 9 or 10 or 11 or 12 or 13 (40507)

15 9 and 10 and 11 and 12 and 13 (0)

16 8 AND 14 {No Related Terms} (11072)

17 training {No Related Terms} (20362)

18 capacity building {No Related Terms} (3142)

19 education {No Related Terms} (12550)

20 guidance {No Related Terms} (8044)

21 teaching {No Related Terms} (15209)

22 tutoring {No Related Terms} (917)

23 supervision {No Related Terms} (4734)

24 17 or 18 or 19 or 20 or 21 or 22 or 23 (64221)

25 8 and 15 and 24 (0)

26 8 or 15 or 24 (64406)

27 referral {No Related Terms} (3453)

28 screening {No Related Terms} (13838)

29 fasting blood sugar {No Related Terms} (11272)

30 FBS {No Related Terms} (7072)

31 random blood sugar {No Related Terms} (3661)

32 RBS {No Related Terms} (1536)

33 glycoslyated hemoglobin {No Related Terms} (3985)

34 glycosylated hemoglobin {No Related Terms} (7266)

35 glycosylated haemoglobin {No Related Terms} (2361)

36 HbA1c {No Related Terms} (6179)

37 HBA1C {No Related Terms} (6179)

38 HbA1C {No Related Terms} (6179)

39 oral glucose tolerance test {No Related Terms} (2662)

40 OGTT {No Related Terms} (8666)

41 Diabetic Retinopathy Screening {No Related Terms} (564)

42 diabetic retinopathy screening {No Related Terms} (564)

43 DR Screening {No Related Terms} (1954)

44 peripheral neuropathy screening {No Related Terms} (679)

45 incidence and prevalence of microvascular complications {No Related Terms} (4515)

46 27 or 28 or 29 or 30 or 31 or 32 or 33 or 34 or 35 or 36 or 37 or 38 or 39 or 40 or 41 or 42 or 43 or 44 or 45 (74358)

47 27 and 28 and 29 and 30 and 31 and 32 and 33 and 34 and 35 and 36 and 37 and 38 and 39 and 40 and 41 and 42 and 43 and 44 and 45 (0)

48 24 and 46 (461)

OVID

1 community {No Related Terms} (39719)

2 health worker {No Related Terms} (6629)

3 1 and 2 (377)

4 lay health worker {No Related Terms} (3986)

5 community health worker {No Related Terms} (1233)

6 CHW {No Related Terms} (1452)

7 4 or 5 or 6 (5764)

8 4 and 5 and 6 (194)

9 diabetes mellitus {No Related Terms} (10248)

10 type 2 diabetes {No Related Terms} (15087)

11 diabetes {No Related Terms} (13755)

12 T2DM {No Related Terms} (3887)

13 DM {No Related Terms} (3275)

14 9 or 10 or 11 or 12 or 13 (40507)

15 9 and 10 and 11 and 12 and 13 (0)

16 8 AND 14 {No Related Terms} (11072)

17 training {No Related Terms} (20362)

18 capacity building {No Related Terms} (3142)

19 education {No Related Terms} (12550)

20 guidance {No Related Terms} (8044)

21 teaching {No Related Terms} (15209)

22 tutoring {No Related Terms} (917)

23 supervision {No Related Terms} (4734)

24 17 or 18 or 19 or 20 or 21 or 22 or 23 (64221)

25 8 and 15 and 24 (0)

26 8 or 15 or 24 (64406)

27 referral {No Related Terms} (3453)

28 screening {No Related Terms} (13838)

29 fasting blood sugar {No Related Terms} (11272)

30 FBS {No Related Terms} (7072)

31 random blood sugar {No Related Terms} (3661)

32 RBS {No Related Terms} (1536)

33 glycoslyated hemoglobin {No Related Terms} (3985)

34 glycosylated hemoglobin {No Related Terms} (7266)

35 glycosylated haemoglobin {No Related Terms} (2361)

36 HbA1c {No Related Terms} (6179)

37 HBA1C {No Related Terms} (6179)

38 HbA1C {No Related Terms} (6179)

39 oral glucose tolerance test {No Related Terms} (2662)

40 OGTT {No Related Terms} (8666)

41 Diabetic Retinopathy Screening {No Related Terms} (564)

42 diabetic retinopathy screening {No Related Terms} (564)

43 DR Screening {No Related Terms} (1954)

44 peripheral neuropathy screening {No Related Terms} (679)

45 incidence and prevalence of microvascular complications {No Related Terms} (4515)

46 27 or 28 or 29 or 30 or 31 or 32 or 33 or 34 or 35 or 36 or 37 or 38 or 39 or 40 or 41 or 42 or 43 or 44 or 45

(74358)

47 27 and 28 and 29 and 30 and 31 and 32 and 33 and 34 and 35 and 36 and 37 and 38 and 39 and 40 and 41 and 42 and

43 and 44 and 45 (0)

48 24 and 46 (461)

EBMR

1. (community health worker* OR community health workers OR CHW OR lay health worker* OR health worker* OR village health worker* OR ASHA OR ANM OR MPHW OR JHA OR MPW OR MPHA)

2. (training OR academic training OR capacity building OR education OR teaching OR supervision OR coaching OR tutorial OR tutoring)

3. (type 2 diabetes mellitus OR blood glucose OR HbA1c OR glycosylated hemoglobin OR fasting blood glucose OR random blood glucose OR glucose tolerance test OR OGTT OR diabetic complications OR microvascular complications)

4. (clinical trial OR randomized controlled trial OR meta-analysis OR systematic review)

5. (low and middle income country OR low and middle income countries OR LMIC OR developing country OR developing countries)

6. 1 AND 2 AND 3 AND 4 AND 5

7. Limits: Publication date from 2000/01/01 to 2024/04/01

WHO ITCRP

Conditions or Disease: Type 2 Diabetes Mellitus, Blood Glucose, HbA1c

Other Terms: Community Health Worker, CHW, Health Worker, Lay Worker

Study Type: Interventional Studies (Clinical Trials)

Study Results: All Studies

Age Group: Adult (18+)

Location: Low and Middle-Income Countries (LMICs)

Study Start From: 01/01/2000

Study End Before: 12/31/2024

Clinicaltrials.gov

Advanced Search Fields:

- **Condition/Disease**: Type 2 Diabetes Mellitus, Blood Glucose, HbA1c
- **Intervention**: Community Health Worker, Lay Worker, CHW, Health Worker
- **Study Type**: Randomized Controlled Trial, Meta-analysis, Clinical Trial
- **Recruitment Status**: All
- **Age Group**: Adults
- **Countries of Recruitment**: Developing Countries, LMIC
