# Supplementary material for: Training Community Health Workers for Diabetes Management in Low- and Middle-Income Countries: Systematic Review
Source: JMIR Diabetes. 2026 Jun 10;11:e84508. doi: 10.2196/84508 (PMC13252886; doi:10.2196/84508)
Supplement: Multimedia Appendix 2 [file diabetes-v11-e84508-s002.pdf]

Annexure 3: Risk of Bias Assessment

Risk of Bias 2 Tool for RCTs

Domain	Jain et al. (2018)	Catley et al. (2022)	de Souza et al. (2017)
**Randomization Process**	Low risk	Low risk	Some concerns
**Deviations from Intended Interventions**	Some concerns	Some concerns	Some concerns
**Missing Outcome Data**	Low risk	Low risk	Some concerns
**Measurement of the Outcome**	Some concerns	Some concerns	Some concerns
**Selection of the Reported Result**	Low risk	Some concerns	Low risk
**Overall Risk of Bias**	Some concerns	Some concerns	High risk

Risk of Bias for Non-RCTs (using ROBINS-I)

Study	Confounding	Selection of Participants	Classification of Interventions	Deviations from Interventions	Missing Data	Measurement of Outcomes	Selection of Reported Results	Overall Bias
Worster et al., 2020	Serious	Moderate	Moderate	Serious	Low	Moderate	Low	Serious
